# Mitochondrial Regulation of Spermatozoa Function: Metabolism, Oxidative Stress and Therapeutic Insights

**DOI:** 10.3390/ani15152246

**Published:** 2025-07-31

**Authors:** Zhiqian Xu, Qi Yan, Ke Zhang, Ying Lei, Chen Zhou, Tuanhui Ren, Ning Gao, Fengyun Wen, Xiaoxia Li

**Affiliations:** 1 College of Animal Science and Technology, Henan University of Science and Technology, Luoyang 471023, China; zqxu@haust.edu.cn (Z.X.); leiying@haust.edu.cn (Y.L.); 9906602@haust.edu.cn (C.Z.); 9906736@haust.edu.cn (T.R.); 2 Henan Provincial Key Laboratory for Grass-Feeding Animals, Luoyang 471023, China; 3 College of Animal Science and Technology, Hunan Agricultural University, Changsha 410125, China; gaon@hunau.edu.cn

**Keywords:** mitochondria, spermatozoa, energy metabolism, reactive oxygen species, mtDNA, antioxidant

## Abstract

Mitochondria are central to energy production and redox regulation in spermatozoa, supporting key functions such as progressive motility, capacitation, and the acrosome reaction. These processes are essential for successful fertilization and embryo development. However, species-specific differences exist in the reliance on oxidative phosphorylation versus glycolysis. Mitochondria also generate reactive oxygen species, which at physiological levels aid in sperm function but can cause oxidative stress and damage when overproduced. Mitochondrial dysfunction and excessive ROS can impair membrane potential, induce apoptosis, and damage nuclear and mitochondrial DNA, ultimately compromising sperm quality. Sperm mitochondrial DNA is highly susceptible to mutations and deletions, contributing to reduced motility and fertility. Targeted antioxidant strategies have emerged as promising therapeutic interventions to mitigate oxidative damage. This article provides a comprehensive overview of mitochondrial regulation in spermatozoa, the consequences of redox imbalance, and the potential of mitochondria-targeted antioxidants to improve sperm function and male fertility outcomes. The paper aims to deepen our understanding of mitochondrial roles in sperm physiology and contribute to the advancement of strategies for addressing male infertility.

## Simple Summary

Male fertility depends greatly on the quality and function of spermatozoa. Spermatozoa need a lot of energy to move and fertilize the egg, and this energy is mainly produced by mitochondria. However, mitochondria can also produce harmful substances called reactive oxygen species, especially when the balance in the cell is disturbed. While small amounts of ROS are necessary for normal sperm function, too much can damage sperm quality. In this review, we summarize how mitochondria produce energy in sperm, how excess ROS can lead to problems, and how damage to mitochondrial DNA can affect sperm health. We also discuss promising treatments by antioxidants that specifically target mitochondria to protect spermatozoa from damage. Understanding how to keep mitochondria healthy in spermatozoa could lead to better treatments for male infertility and help improve reproductive success in both humans and animals.

## 1. Introduction

Infertility affects approximately 10% to 15% of couples worldwide, with male factors contributing to nearly 50% of cases. Male infertility is most commonly associated with poor semen quality, including reduced sperm count (e.g., oligozoospermia or azoospermia), decreased motility (asthenozoospermia), abnormal morphology (teratozoospermia), and increased DNA fragmentation [1]. In livestock breeding, male fertility is also a key determinant of reproductive success, influencing artificial insemination efficiency and overall productivity. For example, the quality of boar spermatozoa significantly affects the conception rate of sows [2,3], and studies have revealed a considerable variation in acrosome integrity among bulls with differing fertility levels [4], indicating the crucial role of sperm quality in determining the reproductive success of male livestock.

Mitochondria play a central role in sperm function by generating ATP through oxidative phosphorylation (OXPHOS), regulating redox balance, and triggering apoptosis [5]. OXPHOS can also generate reactive oxygen species (ROS) as natural byproducts, linking energy metabolism to oxidative stress (OS) regulation. However, excessive ROS production or insufficient antioxidant defenses can disrupt mitochondrial redox balance and lead to sperm damage [6,7]. Mitochondrial ROS are also involved in the fertilization processes such as sperm maturation, capacitation and acrosome reaction. Mitochondrial dysfunction can trigger intrinsic apoptotic pathways, leading to loss of mitochondrial membrane potential (MMP) and the activation of caspases, ultimately compromising sperm viability and fertilization potential. Additionally, mitochondrial DNA (mtDNA) lacks histone protection, making it more susceptible to oxidative damage from ROS, which can lead to mutations that impair OXPHOS and reduce sperm motility [8].

Although several studies have reviewed some aspects of mitochondria in sperm, a comprehensive synthesis that integrates species-specific energy metabolism strategies, mitochondrial redox imbalance, mtDNA vulnerabilities, and therapeutic interventions remains lacking. The article aims to give a comprehensive summary of sperm mitochondrial energy metabolism, outlining the key factors that affect it, discussing how mitochondrial ROS and mtDNA damage contribute to sperm dysfunction, and highlighting current advances in mitochondria-targeted antioxidants as a potential strategy to improve sperm quality and male fertility. This may help to enrich our understanding of mitochondria’s function in sperm, expanding our knowledge of male reproductive physiology, and inspiring new research ideas for addressing male infertility, as well as enhancing the reproductive performance of male animals.

## 2. Relevance of Mitochondrial Energy Metabolism in Spermatozoa

### 2.1. The Mitochondrial Energy Metabolism in Spermatozoa

Spermatozoa produce ATP mainly through glycolysis and OXPHOS [9]. Glycolysis occurs in the fiber sheath of sperm flagella [9,10], converting glucose into pyruvic acid. This process mainly involves a series of enzyme-catalyzed reactions, including hexokinase, phosphofructokinase, and glyceraldehyde-3-phosphate dehydrogenase, etc. [11]. Glycolysis is important in sperm ATP production; inhibition of glycolysis not only reduces ATP levels but also decreases protein tyrosine phosphorylation, a critical signaling event associated with sperm capacitation and hyperactivated motility [12]. Studies have shown that mouse spermatozoa primarily produce ATP by glycolysis [13]. In the medium mainly constituted of glucose, mouse spermatozoa are capable of producing a considerable amount of ATP, which is crucial for maintaining their motility. Notably, any disruption to glycolysis severely impairs mouse sperm motility, even when provided with alternative substrates necessary for OXPHOS [14]. And also, Takei et al. analyzed the distribution of adenosine monophosphate (AMP) in mouse sperm flagella, they found that glycolysis may transport ATP from mitochondria to the distal end of the flagella, thereby maintaining ATP concentration at the distal end of the flagella, which means that glycolysis could also play a role in energy transfer in mouse spermatozoa [15]. Glycolysis is also important in the energy metabolism of human spermatozoa [11,16]. Nascimento et al. performed inhibition treatments targeting both OXPHOS and glycolysis in human spermatozoa, which indicated that OXPHOS did not yield enough ATP to maintain sperm motility, and motility decreases progressively if glycolysis is inhibited [17]. And also, the hyperactivation of human spermatozoa is marked by a requirement for glycolytic substrates such as glucose or fructose, highlighting that the glycolysis pathway is the predominant energy supplier for human sperm function [18]. In the realm of livestock species, the energy metabolism of spermatozoa in ram is similar to that of mice and humans; the inhibition of the glycolysis pathway in ram spermatozoa would result in a decrease of sperm motility and compromised fertilization ability [19]. These findings indicate the critical role of glycolysis across these species.

Another pathway for ATP production is OXPHOS, a mitochondria-dependent process in which ATP is generated via electron transfer through the respiratory chain complexes I–IV and ATP synthase. In this process, NADH^+^, H^+^ and FADH_2_ are oxidized through a series of enzyme-catalyzed reactions and a continuous electron transfer, resulting in the production of H_2_O and ATP [20]. The glycolysis and OXPHOS pathways jointly contribute to ATP production in spermatozoa, but the primary means of ATP production differs among species. Davila et al. inhibited OXPHOS in stallion spermatozoa; the intervention led to a notable reduction in sperm viability, motility, and membrane integrity, illustrating the crucial role of OXPHOS in ATP synthesis for stallion spermatozoa [21]. And also, the preference for OXPHOS over glycolysis in stallion sperm has been confirmed by the Agilent Seahorse XFp Technology [22]. Intriguingly, there are studies that show that stallion spermatozoa also require glycolysis to maintain high sperm velocities, suggesting that stallion sperm not only rely on OXPHOS for the production of ATP but also maintain the capacity to generate ATP via glycolysis [23,24]. Bovine spermatozoa are capable of harnessing both OXPHOS and glycolysis for energy production [25]. Bulkeley et al. have demonstrated that impeding the electron transport chain (ETC) resulted in a marked decline in both the motility and vitality of bovine spermatozoa [26], which highlights the significance of OXPHOS in ATP provision for these cells. Nevertheless, in circumstances where ample glycolytic substrates are available, bovine spermatozoa retain the ability to produce ATP via the glycolytic pathway, showing a flexible energy metabolism in these cells [27]. In addition, pig spermatozoa were thought to predominantly rely on glycolysis for ATP production [28,29], while Prieto et al. found that mitochondrial OXPHOS is the primary source of ATP in fresh boar semen, however, with extended storage under in vitro conditions, the OXPHOS capacity of pig spermatozoa progressively deteriorates, which shifts their energy production towards an enhanced reliance on glycolysis to sustain ATP synthesis [30]. This indicates a temporal adaptation of energy metabolism in pig spermatozoa in response to extrinsic storage conditions. Spermatozoa differ significantly from somatic cells in terms of energy metabolism. While most somatic cells rely predominantly on mitochondrial OXPHOS for steady ATP production [31], sperm cells demonstrate a compartmentalized and species-dependent balance between glycolysis and OXPHOS. Exploring the intricacies of sperm energy metabolism not only elucidates these disparities but also paves the way for refining semen preservation methodologies, thereby contributing to advancements in reproductive science and technology.

### 2.2. Proteins Affecting Mitochondrial Energy Metabolism in Spermatozoa

Many proteins are involved in the spermatozoa energy metabolism process [32]. The oxidative respiratory chain, mainly composed of respiratory chain protein complexes I–IV, ATP synthase, cytochrome C, and coenzyme Q10, is located on the inner membrane of mitochondria and serves as the main site for ATP production and plays a key role in mitochondrial energy metabolism [33,34]. Complex I, which has been known as NADH dehydrogenase, primarily catalyzes the oxidation of NADH within mitochondria [35]. It also serves as a proton pump that transports protons from the matrix into the intermembrane space as electrons pass through, thus forming a proton gradient, and then drives ATP synthase to produce ATP from ADP and inorganic phosphate [36]. Complex II, also known as succinate dehydrogenase (SDH), consists of four subunits (SDHA, SDHB, SDHC, and SDHD) [37]. This complex could transfer electrons from succinate to ubiquinone via its iron-sulfur (Fe–S) clusters, and oxidizes succinate to fumarate in the tricarboxylic acid (TCA) cycle [37,38,39], which is important in reprogramming of metabolic and respiratory adaptation. Complex III is the cytochrome reductase complex, which is responsible for transferring electrons to the cytochrome C receptor [40]. Complex IV is a cytochrome oxidase complex that serves as the final component of the respiratory chain by converting oxygen molecules into water molecules [41]. Complex III and complex IV also possess the function of proton transfer [40], which generates a transmembrane potential that promotes ATP synthase for ATP synthesis [42]. Research has revealed that oligozoospermia patients exhibit significantly lower expression levels of mitochondrial cytochrome oxidase in their spermatozoa compared to normal individuals [43,44], suggesting a direct correlation between the complex III and IV function and sperm quality. Additionally, ATP synthase, a component of the mitochondrial respiratory chain, converts ADP and Pi into ATP by harnessing the transmembrane proton electrochemical gradient created by the electron transfer chain [45,46,47]. Coenzyme Q10, known as ubiquinone, can promote energy production and neutralize the ROS in sperm mitochondria. Deficiency of coenzyme Q10 can lead to decreased sperm motility [48,49,50]. These results demonstrated that the mitochondrial respiratory chain is indispensable in sperm function; impaired mitochondrial respiratory chain protein complexes would destroy sperm energy metabolism.

Besides the respiratory chain proteins, many other proteins have been confirmed to participate in the sperm mitochondria’s energy metabolism. We summarized these proteins and their function in energy metabolism in Table 1. Studying the effects of these molecules on sperm metabolism may help us to better understand the physiological processes of sperm energy metabolism.

## 3. Effects of Mitochondrial ROS on Spermatozoa

### 3.1. Sources of Mitochondrial ROS in Spermatozoa

Mitochondria are the primary source of ROS produced in spermatozoa [65]. The main types of mitochondrial ROS are O^2−^, ·OH and H_2_O_2_; they are oxygen-containing substances with high activity [66]. Mitochondrial ROS primarily occurs during OXPHOS in the inner membrane of mitochondria [67]. Spermatozoa possess antioxidant mechanisms that help maintain ROS balance. Controlled levels of ROS regulate physiological functions and support normal sperm performance, while excessive ROS can cause OS, leading to sperm damage and reduced sperm quality. ROS production in sperm mitochondria is influenced by various factors [6,68]. Spermatogenesis, which includes mitosis, meiosis, and cell differentiation, significantly contributes to ROS generation [69]. The energy metabolic activity of mitochondria also produces substantial ROS as a byproduct [70]. Under stressful conditions, the electron transport during mitochondrial OXPHOS is imperfect, resulting in the formation of superoxide anion (O^2−^). These superoxide anions are then converted into hydrogen peroxide (H_2_O_2_) through the action of superoxide dismutase [66]. Both superoxide anions and hydrogen peroxide have short half-lives and are harmless to sperm under normal circumstances. For example, leukocytes and immature or morphologically abnormal spermatozoa in semen contribute significantly to ROS generation, exacerbating OS and impairing mitochondrial function [71]. Additionally, diseases, nutrient deficiencies, unhealthy lifestyle habits, and environmental pollution are notable contributors to ROS production [72].

### 3.2. Consequences of Mitochondrial ROS Overproduction and Redox Imbalance on Sperm Quality

ROS-mediated damage compromises the structural and functional integrity of spermatozoa [69]. The mitochondrial ETC is involved in the phosphorylation of ADP into ATP [73]. Notably, ROS are primarily generated in respiratory complexes I and III of the ETC [74], highlighting their direct impact on mitochondrial energy metabolism. Indeed, elevated ROS levels have been shown to negatively correlate with the activity of ETC complexes and sperm motility [75,76]. Excessive ROS can reduce cytochrome C oxidase activity in spermatozoa, causing impaired mitochondrial ATP synthesis and disrupted energy metabolism [77]. Excessive ROS also causes lipid peroxide (LP), damaging the polyunsaturated fatty acids (PUFA) in spermatozoa [78], and resulting in mitochondrial electron transport dysfunction and disruption of MMP.

Apart from affecting ATP production, ROS affect the molecular components in spermatozoa. Spermatozoa carry a variety of macromolecules, among which proteins and lipids are especially susceptible to ROS [69] (Chianese and Pierantoni, 2021). ROS regulates the sperm proteins, especially those within the nucleus. ROS can oxidize nuclear proteins in sperm, particularly protamines, which are essential for compacting DNA during spermatogenesis. Oxidative modifications, such as carbonylation or disulfide bond disruption, can impair chromatin packaging and compromise DNA integrity, potentially affecting fertilization and embryo development [79,80]. ROS also increases sperm histone methylation and impairs histone acetylation, resulting in double-stranded DNA breaks, reduced sperm quality, and epigenetic dysregulation [81]. Additionally, some enzymes involved in ATP production and ion channel regulation, as well as sperm proteins modified by tyrosine nitration, exhibit physiological or pathological effects that are closely linked to the levels of ROS generated [82]. In spermatozoa, disulfide bonds exist between cysteine residues of protamine, which contribute to the stability of chromatin. Moderate levels of mitochondrial ROS can facilitate the formation of disulfide bonds, which ensure chromatin stability, protecting DNA from damage, and safeguarding mitochondria against proteolytic hydrolysis [83]. Subsequently, ROS can activate adenylate cyclase and induce intracellular cyclic adenosine monophosphate (cAMP) production, which in turn activates protein kinase A (PKA), extracellular signal-regulated kinase (MEK)-like proteins, threonine-glutamic acid-tyrosine, and fibronectin to enable spermatozoa to achieve final capacitation [84,85,86]. The capacitated spermatozoa undergo phosphorylation of tyrosine proteins, calcium influx, leading to an increase in intracellular cAMP and PKA levels. This triggers the release of proteolytic enzymes, which enable sperm to penetrate and fuse with the egg [83]. Thus, it can be seen that ROS is involved in sperm maturation, capacitation and acrosome reaction, and plays an important role in these processes.

**Figure 1:**
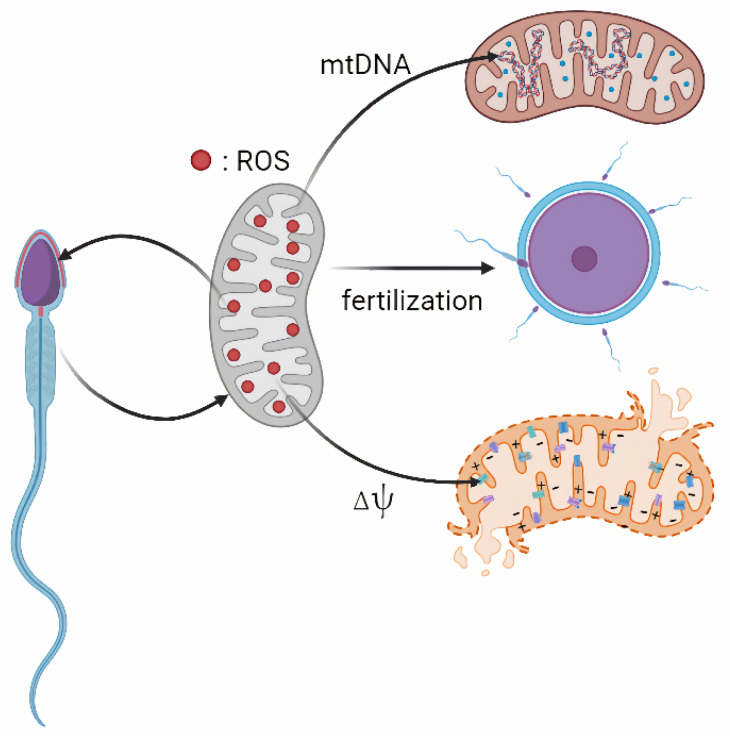
Effects of mitochondrial ROS on spermatozoa. Note: Mitochondria are the major source of ROS in sperm. Excessive ROS can damage sperm MMP (Δψ), as well as sperm nuclear DNA and mtDNA damage. At the same time, ROS also plays an important role in sperm maturation, capacitation and acrosome reaction.

Excessive mitochondrial ROS can also cause epigenetic alterations, telomere shortening, Y chromosome microdeletions, and the activation of apoptotic pathways [87,88]. Due to the limited DNA repair capacity in sperm cells, DNA damage often remains unrepaired, leading to increased phosphorylation and activation of p53, which in turn triggers the mitochondrial-dependent apoptotic pathway [89]. This process also induces the opening of a permeability transition pore, the extrusion of cytochrome c and the activation of a caspase cascade, ultimately resulting in apoptosis-like phenomena [90]. The resulting apoptosis helps eliminate structurally or functionally compromised sperm, thereby preserving overall semen quality. While apoptosis is essential for maintaining sperm homeostasis, excessive activation can contribute to subfertility by reducing the viable sperm population [87]. 8-hydroxy-2′-deoxyguanosine (8-OHdG) is a key indicator reflecting DNA OS damage [91]. 8-OHdG induced incomplete base pairing, which disrupts the ribose-phosphate backbone of DNA and contributes to DNA fragmentation [92]. Comparing the normal fertile men with those experiencing infertility revealed that higher levels of 8-OHdG were found in the infertile group. This elevation suggests that ROS induces oxidative damage to sperm DNA, potentially contributing to male infertility [93,94]. And also, telomeric DNA, composed of guanine-rich repeat sequences, is highly vulnerable to oxidative radical attacks, which can impede the repair and elongation processes of telomeres [95,96]. Additionally, the ROS-induced DNA mutations may transfer and exert effects on the offspring [97,98]. The effects of mitochondrial ROS on spermatozoa have been illustrated in Figure 1.

## 4. The Importance of mtDNA Stability in Mitochondrial Function Maintenance

### 4.1. The Characteristics of Sperm mtDNA

Sperm mtDNA possesses distinct genetic characteristics, notably its maternal inheritance pattern [99]. Both humans and animals have evolved a series of mechanisms to eliminate sperm mitochondria and mtDNA. As a result, the number of mitochondria and mtDNA in spermatozoa is progressively reduced during spermatogenesis and fertilization [100]. Consequently, mtDNA primarily functions in the metabolic processes of spermatozoa before fertilization. And also, the susceptibility to mutation of mtDNA is notable [101]. Sperm mtDNA has a smaller molecular weight and lacks introns; they are in a constant state of synthesis throughout the entire cell cycle, making it less stable and more susceptible to interference from various factors [102]. What is more, mtDNA replication enzymes have poor proofreading capabilities, leading to a higher probability of errors during the duplication process. And the absence of effective repair systems contributes significantly to an escalated risk of mtDNA mutations, which stands at multiple-fold higher frequencies compared to those observed in nuclear DNA [103]. In addition, mtDNA lacks protection from histones and DNA-binding proteins, leaving it directly exposed to the mitochondrial matrix. This makes it susceptible to damage from ROS, leading to a decline of normal physiological functions such as sperm motility, as we described above [104].

### 4.2. Effects of mtDNA Mutation on Sperm Function

The mtDNA abnormalities associated with male infertility have gained much attention [105]. MtDNA mutations in spermatozoa can be generally categorized into point mutations, insert/deletion mutations and mtDNA copy number variation, all of which are known to adversely affect sperm motility and morphology, impairing sperm function and leading to male infertility [106]. There is increasing evidence suggesting that single-nucleotide polymorphisms (SNPs) in mtDNA may significantly influence male fertility [107]. For instance, high levels of A3243G point mutation in mtDNA are strongly linked to decreased sperm motility [108]. And also, some SNPs in mtDNA genes, such as MT-CYB, MT-CO3 and MT-ATP6, have been proven to be associated with male fertility [105,109].

In addition, there are large deletion mutations in mtDNA, which mostly occur in gene fragments with tandem repeat sequences on both sides [102]. The presence of this specific mtDNA deletion in spermatozoa is linked with various male infertility conditions [110]. A higher frequency of 4977, 7599 and 7491 bp mtDNA deletion mutations is associated with asthenozoospermia, asthenoteratospermia and oligasthenospermia in males [111,112,113]. The 4977 bp deletion in mtDNA is among the most extensively researched genetic mutations in spermatozoa. The spermatozoa harboring this mutation show significantly lower motility and may also display abnormalities in morphology, such as head deformities or tail twisting, which render them incapable of successfully fertilizing the eggs [114]. The frequency of spermatozoa with 4977 bp deletion mutation in mtDNA was found to be negatively correlated with the fertilization rate during IVF [115], and the occurrence rate of the 4977bp deletion mutation in mtDNA was higher in males with asthenospermia, oligospermia, and primary infertility [116]. For example, the rate of 4977 bp deletions mutation in patients with asthenospermia was 85.93%; whereas the deletion rate was 14% in normal controls, with a significant difference between the two groups [113]. What is more, a comparative study of 60 infertile men and 60 healthy controls has shown significant associations between the 4977 and 7599 bp deletion mutations and male infertility [112]. These findings indicated that the 4977 bp deletion mutation in sperm mtDNA is significantly higher in male infertility patients compared with the normal group. This prompts the consideration that the 4977 bp deletion mutation has the potential to become a biomarker of sperm fertility. What is more, the deletion mutation resulted in functional defects of respiratory chain proteins encoded by sperm mtDNA, and then impaired ATP generation. This, in turn, can increase the production of mitochondrial ROS or free radicals, ultimately damaging sperm mtDNA [117].

MtDNA copy number is recognized as a sensitive biomarker of mitochondrial integrity and function. In spermatozoa, abnormal mtDNA has been implicated in defective spermatogenesis, heightened mitophagy, and the accumulation of dysfunctional mitochondria. Elevated sperm mtDNA copy number has also been correlated with impaired semen quality, including reduced concentration, total count, viability, and abnormal morphology, and is significantly associated with an increased risk of clinical infertility, Nguyen et al. have summarized the quantitative variations in mitochondrial content and mtDNA copy number across different semen quality categories in various species [7,118]. Importantly, mtDNA copy number in sperm exhibits strong diagnostic potential for predicting male infertility across clinical evaluations [119]. However, investigations into the specific types of sperm mtDNA mutations and their associations with sperm functionality remain limited. Further studies are needed to elucidate the mechanisms through which mtDNA mutations regulate sperm fertilization.

## 5. Mitochondria-Targeted Antioxidant Therapeutic Approaches That Improve Sperm Quality

Given that mitochondria are the origin of ROS, antioxidant-based strategies targeting mitochondria have emerged as promising interventions to restore redox balance and improve sperm function. The common mitochondria-target antioxidants include ubiquinone (MitoQ), melatonin, quercetin, MitoTEMPO, etc. Mitochondrial-target antioxidants block oxidative damage through covalent attachment of lipophilic cations such as lipophilic cation triphenylphosphonium (TPP^+^) in mitochondria [120]. The TPP^+^ of mitochondrial-target antioxidants can easily penetrate the mitochondrial membrane’s lipid bilayer and deliver the antioxidant to the mitochondrial matrix [121]. Once inside the mitochondria, these compounds can accumulate in large amounts, exhibiting significant mitochondrial antioxidant effects [122].

MitoQ is a widely studied mitochondrial-target antioxidant [123]. It accepts electrons from mitochondrial respiratory chain protein complex I or II, and reduces to ubiquinol, which then transfers these electrons to mitochondrial complex III [122]. Ubiquinol also acts as an antioxidant by providing hydrogen atoms to lipid peroxyl radicals, thereby preventing LP [124]. Supplementation of MitoQ in human spermatozoa resulted in improved sperm function, attenuated DNA damage, and concurrently induced the upregulation of antioxidant gene expression, thereby conferring supplementary benefits for sperm cryopreservation [125]. Studies have been conducted to assess the comparative effects of MitoQ and a cytoplasmic antioxidant RESV on buffalo semen subjected to cryopreservation. Results showed that MitoQ significantly improved sperm motility and viability prior to freezing compared to RESV (*p* ≤ 0.05), and MitoQ-treated sperm displayed heightened acrosome integrity and a greater proportion of viable spermatozoa post-freezing and thawing [126]. Furthermore, MitoQ treatment was found to boost the motility of freshly collected fish sperm and decrease LP levels; it may be attributed to its effective reduction of ROS generation in fish sperm following the freeze-thaw process [127]. These results indicate that MitoQ exerts a mitochondrial-targeted antioxidant effect and can mitigate ROS-induced sperm cryodamage in frozen-thawed sperm.

Melatonin is also an antioxidant that targets mitochondria [128]. Melatonin is predominantly synthesized within mitochondria and exerts its principal effects [129]. Melatonin maintains optimal MMP by scavenging ROS, activating uncoupling proteins, and inhibiting 1 methyl-4-phenyl-1,2,3,6- tetrahydropyridine formation [130]. Melatonin can also optimize the distribution of enzymes required for OXPHOS in spermatozoa, enhancing the activity of respiratory chain protein complexes, ultimately boosting the oxygen utilization capacity of cryopreserved spermatozoa upon thawing [131]. In human spermatozoa, melatonin can improve mitochondrial function by reducing mitochondrial OS [132]. And melatonin demonstrates a protective effect against environmental toxicity and cold-induced damage [133]. The addition of melatonin at varying concentrations has been shown to enhance sperm viability and motility, concomitant with a decrease in intracellular ROS levels. However, the optimal melatonin concentration for these beneficial effects varies across different studies [134,135,136]. Moreover, melatonin supplementation in cryopreservation medium for farm animals such as bovine [137], rams [138], buffalo [139], and pigs [140] has produced similar positive outcomes. Thus, melatonin supplementation plays a protective role in safeguarding both human and livestock spermatozoa, preserving their viability, and diminishing abnormal morphology and DNA fragmentation during the cryopreservation process. As a result, it has gained widespread application as an antioxidant additive in sperm cryopreservation [124,141].

Quercetin is a mitochondria-targeted flavone; it can reduce lipid peroxide accumulation by neutralizing harmful free radicals and attaching to transition metal ions. This activity alters the fluidity of the mitochondrial membrane and affects the oxidative proteins within the mitochondrial matrix [142,143]. It also modulates mitochondrial biogenesis, MMP, OXPHOS and ATP production, mitochondrial redox states, and ultimately triggers mitochondria-mediated apoptosis [144]. Quercetin could modulate sperm mitochondrial respiration efficiency in asthenozoospermic men [145], and decreases ram sperm motility within the first two hours by acidifying the incubation medium, while subsequently stimulates sperm motility during the following three to four hours by maintaining mitochondrial respiration [146]. Quercetin is also beneficial for spermatozoa during cryopreservation due to its antioxidative properties, as the freezing and thawing process typically induces high levels of OS. The antioxidant capability of quercetin has been confirmed across various animal species, demonstrating its efficacy in safeguarding sperm from oxidative stress during the cryopreservation procedure [147]. The addition of quercetin to the freezing solution was found to decrease oxidative damage and thereby enhance the sperm quality after thawing in human [148], pig [149], goat [150], rooster [151], dog [152] and so on. Despite its benefits, the precise molecular mechanisms behind quercetin’s actions are yet to be fully understood, and there are also studies showing that quercetin has no significant effects on frozen sperm quality. Further research is necessary to elucidate its protective effects in semen cryopreservation.

MitoTEMPO is a ROS scavenger composed of piperidine nitroxide TEMPOL and lipophilic triphenylphosphine (TPP). As a cell-permeable novel antioxidant, its lipophilic properties enable it to rapidly permeate through the lipid bilayer membrane of mitochondria and accumulate in high concentrations within these organelles [153]. Research has demonstrated that MitoTEMPO selectively targets mitochondrial superoxide, thereby enhancing mitochondrial function and the cell’s antioxidant capabilities [154]. Currently, research on MitoTEMPO on sperm quality is predominantly centered on semen cryopreservation. This emphasis is placed because OS has been recognized as a key factor affecting sperm quality, which could lead to the increase of ROS and LP during the cryopreservation process [155]. Research has shown that the addition of MitoTEMPO significantly enhanced the human post-thaw sperm motility, membrane integrity and MMP, and reduced the ROS level in sperm mitochondria, suggesting that MitoTEMPO could serve as an effective cryoprotectant for semen samples, and also, MitoTEMPO could alleviate cryodamage of asthenozoospermic spermatozoa after cryopreservation [155,156,157]. Additionally, research has extended to examine the impact of MitoTEMPO on post-thaw spermatozoa from various animal species, including bulls [158,159], rams [160,161,162,163], roosters [164,165], and tomcats [166]. These studies revealed that supplementation of MitoTEMPO significantly improved sperm quality in comparison to the untreated control group. However, further studies are required regarding MitoTEMPO’s impact on non-cryopreserved semen, as well as its effects on sperm quality in species such as pigs. Moreover, in-depth exploration of the mechanisms by which MitoTEMPO influences sperm quality can enhance our comprehension of sperm antioxidant systems, thereby facilitating the discovery of more efficacious mitochondria-targeted antioxidants to boost sperm quality.

The mitochondrial-target antioxidants have gained considerable attention, highlighting the critical role of mitochondrial antioxidant activity in maintaining sperm function. However, their effects have yielded inconsistent results, which may be explained by a biphasic, concentration-dependent response of sperm cells to the antioxidants. In addition to the previously introduced mitochondria-targeted antioxidants, an increasing number of compounds with mitochondrial antioxidant properties have also been investigated for their roles in sperm function. It can be deduced that mitochondria-targeted antioxidants are potentially useful in ameliorating OS in spermatozoa, particularly demonstrating favorable outcomes in mitigating mitochondrial OS induced by cryopreservation. Future research and endeavors should be taken to examine the effectiveness of mitochondrial antioxidants, elucidating the underlying mechanisms of their action, and developing additional potent mitochondria-specific antioxidants. Table 2 provides a summary of studies that have investigated the use of mitochondria-targeted antioxidants in spermatozoa.

## 6. Conclusions

Mitochondria play a central role in regulating sperm energy metabolism, redox balance, and overall function. The role of mitochondria in sperm energy metabolism varies across species, and proteins within sperm are among the key molecules influencing mitochondrial function. While physiological levels of mitochondrial ROS are essential for normal sperm processes such as capacitation and acrosome reaction, excessive ROS production leads to oxidative stress, mitochondrial dysfunction, and damage to nuclear and mitochondrial DNA. Mitochondria-targeted antioxidants have shown promising results in preserving sperm motility, viability, and fertilization potential by restoring redox balance and protecting mitochondrial integrity. However, more research is needed to optimize antioxidant type, dosage, and delivery methods across species. Future studies should also focus on refining diagnostic tools for assessing mitochondrial health in sperm and evaluating antioxidant strategies in clinical and agricultural settings. Such approaches may provide effective interventions for improving male fertility and reproductive efficiency.

## Figures and Tables

**Table 1:** Sperm proteins are involved in mitochondrial energy metabolism.

Protein	Species	Function	Ref.
Ant2	Drosophila	Disrupting mitochondrial morphogenesis and spermatid maturation	[51]
MORN2	Mouse	MORN2 functions in mitochondrial sheath formation and regulates mitochondrial respiratory activity	[52]
ClpP/ClpX	Mouse	ClpP/ClpX deficiency disrupted energy supply during meiosis and impaired zygotene-pachytene transformation in male germ cells	[53]
Rsrc1-161aa	Mouse	Regulating mitochondrial ribosome assembly and translation, and then affecting male fertility.	[54]
SLC22A14	Mouse	Shortage of SLC22A14 could decrease the energy metabolism of spermatozoa	[55]
PRSS55	Mouse	Deficiency of PRSS55 damages the mitochondrial structure, which leads to lower ETC molecules and ATP contents	[56]
SLC7A11 antiporter	Stallion	Transporting cystine into the cell in exchange for glutamate	[57]
DJ-1	Rat	Interacting mitochondrial complex I subunits NDUFS3 and NDUFA4 affect mitochondrial energy metabolism	[58]
GSK3	Goat	Phosphorylation of GSK3 regulated sperm motility and acrosome reaction by glycolysis and OXPHOS	[59]
TPPP2	Mouse	Deficiency of TPPP2 altered the structure and function of sperm mitochondria and caused decreased sperm count, motility and ATP content	[60]
AMPK	Mouse/Goat	Increasing AMPK activity maintains MMP and ATP levels in spermatozoa	[61,62]
LDHC	Mouse	Maintenance of energy metabolism in sperm	[63]
GLUT8	Mouse	Lack of GLUT8 leads to impaired mitochondrial function and sperm motility in spermatozoa	[64]

Note: Ant2 (Adenine nucleotide translocase 2), MORN2 (nexus-motif protein 2), ClpP (Caseinolytic protease proteolytic subunit), ClpX (caseinolytic protease X), PRSS55(serine protease 55), AMPK (AMP-activated protein kinase), MMP (mitochondrial membrane potential), GSK3 (Glycogen synthase kinase-3), TPPP2 (tubulin polymerization promoting protein family member 2), LDHC (lactate dehydrogenase C).

**Table 2:** Applications of mitochondria-targeted antioxidants on sperm quality.

Antioxidants	Species	Functions	Ref.
MitoQ	Rooster	Improve the sperm quality in cryopreserved and cooled rooster spermatozoa	[167,168]
	Yak	Increase the sperm quality and antioxidant capacity in low-temperature yak semen preservation	[169]
	Human	Enhance functional sperm parameters, reduce DNA fragmentation, and upregulate the expression of antioxidant-related genes in human sperm cryopreservation medium	[125,170]
	Stallion	Improve the frozen-thawed stallion sperm motility and kinetics, while detrimental to the motility and viability at higher concentrations	[171]
	Buck	Improve the motility, viability, membrane functionality, mitochondrial activity, acrosome integrity, and reduce OS during the cryopreservation process of buck sperm	[172]
	Bull	Do not affect the Simmental bull sperm cryo-resistance	[173]
		Enhance post-thaw buffalo bull sperm function and cryo-tolerance	[126]
	yellow catfish	Reduce ROS production and LP, and enhance post-thaw sperm viability of yellow catfish	[127]
Melatonin	Mouse	Alleviate post-weaning NaAsO2 exposure-mediated decline of sperm counts in adult mice	[133]
	Human	Improve human sperm quality and overall male fertility	[132]
		Counteract intracellular ROS and MDA, increase post-thaw viability, motility and membrane integrity of human spermatozoa	[134,135,136]
	Ram	Improve OXPHOS of frozen-thawed ram sperm	[131]
		Improve progressive motility and reduce DNA fragmentation of ram spermatozoa following cryopreservation	[138]
	Bull	Improve sperm function parameters in the fresh and post-thaw mithun bull semen	[137]
		Improve sperm viability and motility of swamp buffalo during cryopreservation	[139]
	Boar	Increase the number of live sperms with intact acrosome, but did not affect the other spermatic quality of boar semen	[140]
Quercetin	Human	Improve the human sperm progressive motility, MMP, and decrease ROS levels, DNA fragmentation	[148]
		Stimulate the respiratory active state and enhance mitochondrial function in asthenozoospermic samples	[145]
	Boar	Maintain sperm motility and decrease superoxide in the liquid preservation of boar semen, and improve the post-thaw sperm quality and reduce the OS of boar sperm	[149,174]
	Canine	Improve the post-thaw sperm quality and reduce the oxidative damage in the dog	[152]
	Goat	Decrease the goat sperm MDA and ROS levels, enhance motility, viability, membrane integrity, and mitochondria activity	[150]
	Rooster	Improve post-thawed sperm quality and fertility of roosters	[151]
	Ram	Inhibit the ram spermatozoa motility during the first 2 h of incubation, but subsequently stimulate motility over the following 3–4 h by sustaining mitochondrial respiration	[146]
MitoTEMPO	Human	Improve post-thaw human sperm motility, viability, membrane integrity, MMP and antioxidant enzymes activities decrease DNA fragmentation index, ROS and MDA level	[155,156,157]
	Bull	Improve post-thaw buffalo sperm progressive motility, viability, acrosomal and membrane integrity, cholesterol to phospholipids ratio, MMP, antioxidant enzymes activities and fertility	[158,159]
	Ram	Improve the post-thaw ram sperm motility, kinematics, viability, membrane functionality, MMP, antioxidant capacity, glucose transporter and fertility, decreasing MDA	[160,161,162,163]
	Rooster	Improve the post-thaw rooster sperm motility kinematics, membrane functionality, MMP, viability and fertility, decrease lipid peroxidation, late apoptotic-like changes, DNA fragmentation and hydrogen peroxide level	[164,165]
	Tomcat	Improve the acrosome integrity of frozen-thawed epididymal spermatozoa in tomcats	[166]
MitoPBN	Ram	Mitigate OS and improve mitochondrial function in cryopreserved ram sperm	[175]
Crocin	Human	Improve human sperm progressive motility, MMP, and decrease ROS levels, DNA fragmentation, enhance the asthenozoospermic sperm motility and reduce OS	[148,176]
	Bull	Improve the sperm post-thaw quality and fertility of buffalo bull and Yanbian yellow cattle sperm	[177,178,179]
	Rooster	Improve the post-thawing rooster sperm viability, membrane functionality, MMP and fertility, reduce sperm LP and apoptosis	[180]
Naringenin	Bull	Improve buffalo bull post-thaw quality, fertility-associated gene expression and fertilization potential	[181]
	Boar	Maintenance of sperm motility and decrease of superoxide in the liquid preservation of boar semen	[174]
	Rooster	Improve the post-thawing rooster sperm viability, membrane functionality, MMP and fertility, reduce sperm LP and apoptosis	[180]
Metformin	Equine	Improve total motility and reduce LP of thawed equine spermatozoa	[182]
	Sheep	Improve the post-thawing sheep sperm quality and reduce sperm OS	[183]
	*S. prenanti*	Improve the quality and fertility of *S. prenanti* sperm by increasing the ATP content	[184]
	Human	Inhibit human sperm motility, PKA and protein tyrosine phosphorylation pathways, do not affect sperm viability, MMP, mitochondrial superoxide anion generation	[185]
	Boar	The effects on boar sperm quality remain controversial	[186,187]
	Canine	Improve the frozen-thawed canine sperm motility, mitochondrial activity, NAD^+^ content, and reduce the OS level	[188]
Rosiglitazone	Equine	Increase the ROS level of thawed equine spermatozoa. Increase motility, MMP, ATP content, glucose uptake capacity, and decrease ROS level of equine spermatozoa in vitro.	[182,189]
	Bull	Maintain MMP, total and progressive motility of bovine sperm	[190]
	Ram	Enhance motility, membrane and acrosome integrity, MMP, and decrease ROS level in frozen-thawed ram spermatozoa	[191]
	Boar	Enhance boar sperm motility, membrane and acrosome integrity, MMP, ATP production and reduce ROS during storage at 17 °C	[192]
Pyrroloquinoline quinone	Boar	Enhance post-thaw boar sperm motility, viability, acrosome integrity, MMP, ATP levels, mtDNA stability, and decrease MDA, ROS levels and DNA damage	[193]
	Ram	Improve sperm motility, MMP, membrane and acrosome integrity, ATP levels and fertility, decrease sperm MDA and ROS levels of ram sperm at 4 °C	[194]
	Bull	Reduce mitochondrial ROS in fresh bull semen, improve sperm motility and reduce ROS in frozen-thawed bull sperm	[195]
Astaxanthin	Rooster	Improve rooster freeze-thaw sperm viability, motility, plasma membrane and acrosome integrity, mitochondrial activities, ATP level and antioxidant ability	[196,197]
	Ram	Increase sperm motility, viability, plasma membrane and acrosome integrity, MMP and antioxidant ability of Hu ram spermatozoa at 4 °C	[198]
	Canine	Increase sperm motility, viability, plasma membrane and acrosome integrity, and mitochondria activity of post-thaw dog spermatozoa	[199]
Ergothioneine	Boar	Improve sperm motility parameters, MMP, ATP, antioxidant capacity, plasma membrane and acrosome integrity in in vitro liquid preservation of boar spermatozoa	[200]
	Bull	Improve sperm motility and reduce ROS level in frozen-thawed bull sperm	[195]
	Canine	Increase the total motility, acrosomal integrity and reduce the abnormal morphology and ROS production of cryopreserved canine spermatozoa	[201]
	Rooster	Increase the total motility, membrane integrity and mitochondria activity, reduce the apoptotic and dead sperm of post-thawed rooster spermatozoa	[202]
Losartan	Rooster	Improve the total and progressive motility, mitochondrial activity, membrane integrity, antioxidant levels, and reduce LP and apoptosis of post-thawed rooster spermatozoa	[203]
Lagenaria siceraria seed oil	Rabbit	Improve sperm motility, viability, membrane integrity and antioxidant capacity during 72-h chilled storage of rabbit spermatozoa	[204]
Urolithin A	Bull	Improve bovine sperm motility quality and reduce OS	[205]
AntiOxBEN2	Bull	Enhance the bovine sperm quality, fertility, and reduce ROS	[206]
BGP-15	Human	Improve sperm motility, mucous penetration and MMP, reduce sperm DNA oxidative damage of post-thawed human spermatozoa	[170]
L-Carnitine	Human	Improve sperm motility, viability, and reduce sperm DNA oxidative damage of post-thawed human spermatozoa	[170]
Cysteine	Ram	Improve the rates of ALH, membrane integrity, and mitochondrial activity of the post-thawed ram sperm	[207]
Isoespintanol	Canine	Increase the acrosomal integrity and reduce the abnormal morphology and ROS production of cryopreserved canine spermatozoa	[201]
Vitamin C	Bull	Reduce mitochondrial ROS in fresh bull semen, improve sperm motility and reduce ROS in frozen-thawed bull sperm	[195]
EGCG@PDA NPs	Boar	Maintain sperm motility, acrosome integrity, MMP, extending semen storage time from 3 days to 10 days, reducing ROS and sperm apoptosis of boar sperm during storage at 4 °C	[208]

## Data Availability

There are no new data associated with this article.
